# A spotlight on the interplay between Wnt/β-catenin signaling and circular RNAs in hepatocellular carcinoma progression

**DOI:** 10.3389/fonc.2023.1224138

**Published:** 2023-07-21

**Authors:** Alireza Mafi, Hamidreza Rismanchi, Mohaddese Malek Mohammadi, Neda Hedayati, Seyedeh Sara Ghorbanhosseini, Seyed Ali Hosseini, Yasaman Gholinezhad, Rohollah Mousavi Dehmordi, Behrooz Ghezelbash, Fatemeh Zarepour, Seyed Pouya Taghavi, Zatollah Asemi, Mina Alimohammadi, Hamed Mirzaei

**Affiliations:** ^1^ Department of Clinical Biochemistry, School of Pharmacy and Pharmaceutical Sciences, Isfahan University of Medical Sciences, Isfahan, Iran; ^2^ Nutrition and Food Security Research Center, Isfahan University of Medical Sciences, Isfahan, Iran; ^3^ School of Medicine, Shahid Beheshti University of Medical Sciences, Tehran, Iran; ^4^ School of Medicine, Iran University of Medical Science, Tehran, Iran; ^5^ Research Committee, Department of Immunology, School of Medicine, Shahid Beheshti, University of Medical Sciences, Tehran, Iran; ^6^ Department of Pharmacology, School of Medicine, Shahid Beheshti University of Medical Sciences, Tehran, Iran; ^7^ Department of Clinical Biochemistry, Faculty of Medicine, Ahvaz Jundishapur University of Medical Sciences, Ahvaz, Iran; ^8^ Department of Immunology, School of Medicine, Isfahan University of Medical Sciences, Isfahan, Iran; ^9^ School of Medicine, Kashan University of Medical Sciences, Kashan, Iran; ^10^ Research Center for Biochemistry and Nutrition in Metabolic Diseases, Institute for Basic Sciences, Kashan University of Medical Sciences, Kashan, Iran; ^11^ Student Research Committee, Department of Immunology, School of Medicine, Shahid Beheshti University of Medical Sciences, Tehran, Iran

**Keywords:** Wnt signaling, circRNA, hepatocellular carcinoma, mechanism, non-coding RNA

## Abstract

Hepatocellular carcinoma (HCC) is one of the deadliest cancers due to multifocal development and distant metastasis resulting from late diagnosis. Consequently, new approaches to HCC diagnosis and treatment are required to reduce mortality rates. A large body of evidence suggests that non-coding RNAs (ncRNAs) are important in cancer initiation and progression. Cancer cells release many of these ncRNAs into the blood or urine, enabling their use as a diagnostic tool. Circular RNAs (CircRNAs) are as a members of the ncRNAs that regulate cancer cell expansion, migration, metastasis, and chemoresistance through different mechanisms such as the Wnt/β-catenin Signaling pathway. The Wnt/β-catenin pathway plays prominent roles in several biological processes including organogenesis, stem cell regeneration, and cell survival. Aberrant signaling of both pathways mentioned above could affect the progression and metastasis of many cancers, including HCC. Based on several studies investigated in the current review, circRNAs have an effect on HCC formation and progression by sponging miRNAs and RNA-binding proteins (RBPs) and regulating the Wnt/β-catenin signaling pathway. Therefore, circRNAs/miRNAs or RBPs/Wnt/β-catenin signaling pathway could be considered promising prognostic and therapeutic targets in HCC.

## 1 Introduction

Hepatocellular carcinoma (HCC) is an invasive type of cancer that leads to over 700,000 deaths annually worldwide (1). This tragedy occurs due to lack of effective diagnostic tools as well as efficient therapeutic approaches (2, 3). Although surgical removal, liver transplants, and chemotherapeutic agents are the most commonly used treatments for HCC, they are ineffective in many cases due to multifocal development and distant metastasis caused by late diagnosis (4, 5). Approximately 70% of patients with HCC are diagnosed late, therefore, even if these therapeutic strategies are used in early stages, they are unable to improve the 5-year overall survival rate over 70% (6, 7). As a result, advances in diagnostic and therapeutic approaches must be expanded to reduce HCC mortality.

Non-coding RNAs (ncRNAs) are RNAs derived from the human genome but not translated into protein (8). NcRNAs regulate gene expression through several pathways, such as interactions with epigenetic factors to silence genes and interactions with transcription factors to inhibit or promote target gene expression (9). Based on abundant evidence, ncRNAs play an important role in the initiation and progression of various cancers (10, 11). Cancer cells release many of these ncRNAs into the blood or urine, so they can be used as a diagnostic factor. These ncRNAs are divided into four main types, including microRNA (miRNA), long non-coding RNA (lncRNA), circular RNA (circRNA) and PIWI interacting RNA (piRNA) (12). CircRNA have a unique and stable structure that comprises covalently closed loops (13). They are crucial in the development of several malignancies, including colorectal cancer (14), cholangiocarcinoma (15), bladder cancer (16), and HCC (17), through the regulation of cancer cell proliferation, migration and metastasis, and chemoresistance (18). Most important functions of circRNA for regulating target gene expression are miRNA regulation and effect on the signaling pathways such as Wnt/β-catenin (19).

The Wnt family includes a group of proteins that play important roles in a various cellular functions, such as organ development, stem cell renewal, and the cell viability (20). The Wnt Cascade is divided into different branches, including the canonical Wnt/β-catenin (Wnt/β-catenin dependent pathway) and the non-canonical Wnt/β-catenin pathway (β-catenin-independent pathway). The latter is further divided into the Wnt/calcium and planar cell polarity (PCP) pathways that have an effect on cancer progression and propagation (21). Wnt/β-catenin is involved in numerous physiological mechanisms, such as cell expansion and differentiation, cell death, migration, and tissue homeostasis (22). Abnormalities in Wnt signaling have been linked to several cancers, including gastrointestinal cancers, especially colorectal cancer, leukemia, melanoma, breast cancer and also in a significant subset of HCC (23, 24).

Several studies have found that circRNAs have the potential to be used as biomarkers in tissue samples for cancers diagnosis, with noticeable clinical applications for predicting HCC prognosis. This could potentially lead to improved therapeutic strategies (25, 26). For instance, Peisi kou et al, revealed that expression of Circular RNA hsa_circ_0078602 was meaningfully decreased in 79 samples of HCC patients when compared to controlled non-tumoral tissues (P=0.015). Kaplan-Meier survival analysis indicated that lower expression of hsa_circ_0078602 is associated with poor prognosis in HCC cases (27). In contrast, another study on HCC tissue samples from 59 patients found that circ_0000267 is associated with poor prognosis and promotes proliferation, migration and infiltration of cancer cells through sponging miR‐646 (28).

Our theory is that circRNAs could become a novel approach for detecting and treating HCC in the future. To support this idea, we conducted a review of recent studies that investigate the impact of circRNAs on HCC development, with a particular focus on how they affect miRNA regulation and the Wnt/β-catenin signaling pathway.

## 2 Wnt pathway: canonical and non-canonical signaling

**Figure 1:**
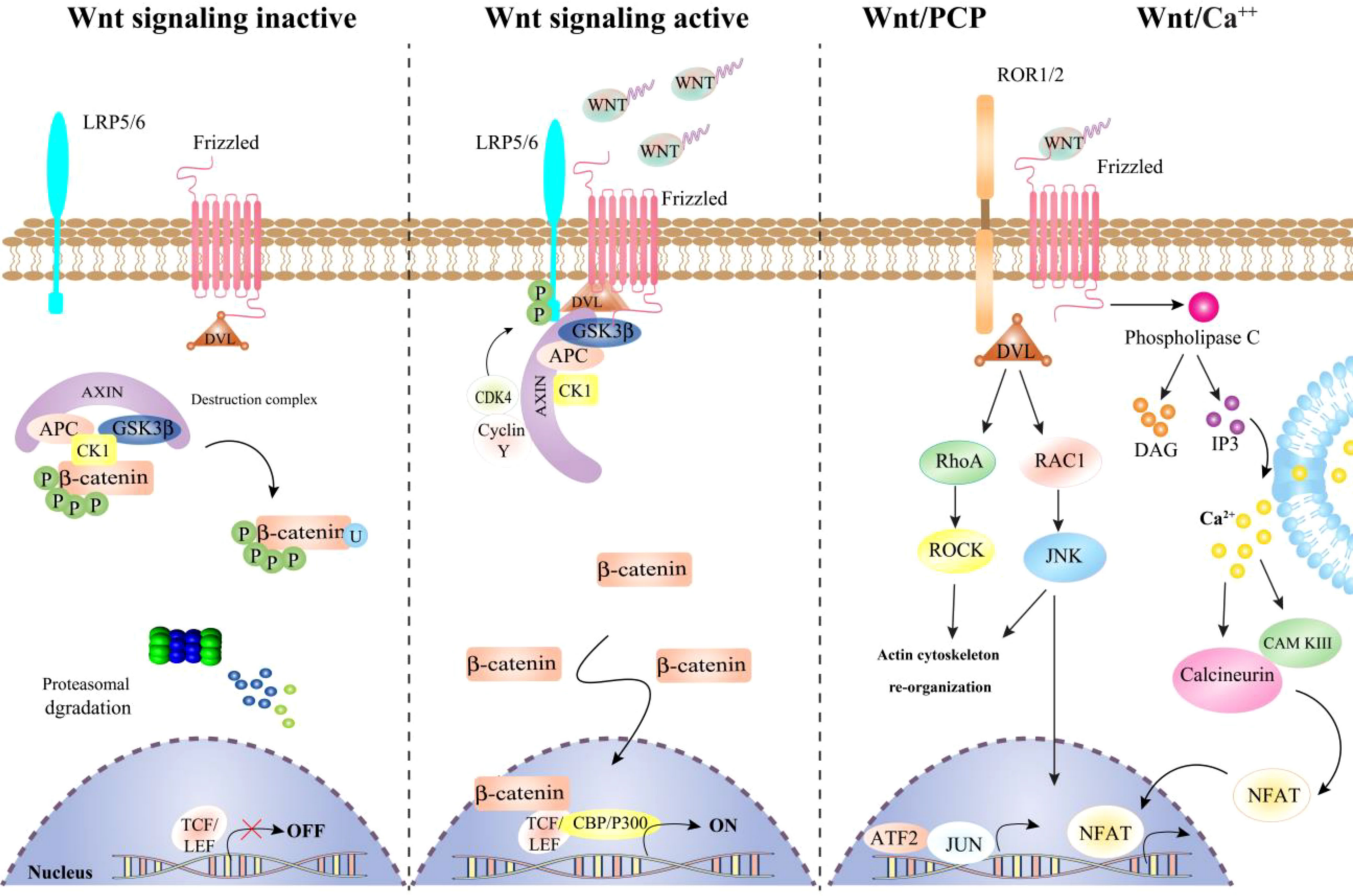
A schema of canonical and non-canonical Wnt signaling pathway.

The Wnt pathway comprises two types of signal transduction pathways (STPs): canonical (Wnt/β-catenin) and non-canonical (planar cell pole (PCP) and Wnt/calcium (Wnt/Ca2+)) (29, 30) as shown in **(** **Figure 1** **)**. Many studies have investigated the role of Wnt signaling in the tumorigenesis, with an focus on signaling that relies on β-catenin (31). In the absence of Wnt, a complex of β-catenin degradation proteins, including Axis inhibition protein (AXIN), adenomatous polyposis coli (APC), and glycogen synthase kinase 3-beta (GSK-3-beta), is formed. This complex breaks down β-catenin through phosphorylation at both serine and threonine residues. When Wnt is present, it binds to one of the ten Frizzled (FZD) receptors, leading to the formation of a receptor complex containing Wnt, FZD, lipoprotein receptor-related protein (LRP), Disheveled (DVL), and AXIN. Inside this dynamic complex, DVL is phosphorylated and eventually blocks GSK-3-beta, which makes β-catenin less phosphorylated and prevents its proteolytic degradation (32). β-catenin is then stored in the cytoplasm and can be translocated to the nucleus, where it interacts with members of the TCF/LEF family of gene-activating transcription factors, such as CREB-binding protein (CBP) and p300. c-Myc and cyclin D1 among the many target genes (33). Receptor tyrosine kinase-like orphan receptor 2 (ROR2), a transmembrane orphan receptor tyrosine kinase required for non-canonical Wnt signaling, can also function as canonical signaling through FZD2 interaction (34). Therefore, ROR2 and other Wnt-binding receptors like the receptor tyrosine kinase RYK may play a regulatory role in β-catenin-independent Wnt signaling. Wnt ligands that activate the two non-canonical Wnt pathways include Wnt4, Wnt5a, Wnt7a, Wnt11, and Wnt16. Wnt11. For instance, stimulates the PCP signaling pathway, which in turn activates the small GTPases RAS homologue gene-family member A (RhoA) and Ras-related C3 botulinum toxin substrate 1 (RAC1) (35). This causes the stress kinases Jun N-terminal kinase (JNK) and Rho-associated coiled-coil-containing protein kinase 1 (ROCK) to be activated, altering cell adherence and migration. Wnt5a is the most well-known Ca2^+^-dependent Wnt signaling inducer, triggering STP via DVL-3 and phospholipases (36). When this pathway is activated, there is a temporary increase in cytoplasmic free Ca2^+^, which can stimulate the PKC family, calcium calmodulin mediated kinase II (CaMKII), and the calcineurin phosphatase. In addition to Ca2^+^-dependent Wnt STP, tumor cells have been found to have a novel non-canonical Wnt STP mediated by FYN tyrosine kinase and STAT3 (37). Wnt proteins are controversial in that they may interact with receptors, control the Wnt STP and Wnt-related elements. For instance, non-canonical Wnt signaling inhibits canonical Wnt activity via a variety of pathways, including PKC-alpha, CaMKII-Transforming growth factor β-Activated Kinase (TAK)1, Nemo-like Kinase (NLK), Siah2 E3 ubiquitin ligase, and calcineurin-NFAT. Non-canonical Wnt ligands have also been shown to activate the canonical pathway and further complicate Wnt signaling, making modification in cancer therapies challenging (38).

## 3 CircRNAs: biogenesis and biological functions

According to the circular structure and lack of 3′-end and 5′-end of circRNAs, they have higher stability than linear RNAs and resist degradation by RNAase R (39, 40). CircRNAs play an essential role in various cellular processes and gene expression via regulating RNA polymerase II function and participating in mRNA processing (41). Thousands of genes have been found to produce circRNAs in mammals. Unlike linear RNAs, circRNAs are generated through the non-sequential back-splicing (or exon skipping) of protein-coding RNA precursors catalyzed by spliceosomes or ribozymes (42). CircRNAs may arise from different gene parts such as exons, introns, intergenic sites, antisense, and untranslated regions (UTRs) (43) and are divided into five major types based on the origin of their gene: Exonic circRN*A* (the most abundant type), intronic circular RNA, Exon-intron circular RNA, Intragenic circular RNAs, and Antisense circular RNAs (39). The two most widely accepted models for exonic-circRNA biogenesis are lariat-induced circularization and intron pairings (44). Non-adjacent exons of a pre-mRNA become closer together in the lariat-driven circularization model by developing an intermediate lariat structure containing multiple exons and introns. Exonic circRNA is produced after intron separation by connecting the downstream exon (splice donor) and upstream exon (splice receptor) (45). In some cases, during lariat-driven circularization, introns are not spliced out, resulting in the formation of exon-intron circRNA (46). The intron pairing-driven circularization is based on the complementary pairing of Alu repeats on the intron ends, followed by pre-mRNA reverse splicing, which results in the formation of exonic and exon-intron circRNA (43). The functions of circRNA generally are: regulating gene splicing and transcription, regulating protein translation and function, sponging miRNA, and interacting with RNA-binding proteins (RBP) (47, 48). Localization of circRNA can also determine its function, as nuclear circRNAs mainly regulate transcriptional processes, and cytoplasmic circRNA mainly regulates the functions of miRNA and protein. Even though most circRNA are ncRNAs, recent studies revealed that some cytoplasmic circRNA can be translated to peptides (49). Expression of circRNA is different in various cell types and tissues; for instance, circRNA is widely distributed in the brain and is involved in brain development and function (50, 51). As circRNAs play a pivotal role in regulating many cellular processes, aberrant expression of circRNA leads to impaired cellular function. Several investigations have revealed that circRNA is overexpressed in a variety of human diseases, including cardiovascular disease, neurologic disease, autoimmune disease, and cancers (52). CircRNA can exert tumorigenesis or tumor suppressor effects through various mechanisms, such as regulating cell proliferation and apoptosis. Therefore, circRNA can be considered a diagnostic marker and therapeutic target in several diseases, including different types of cancers.

## 4 CircRNAs in HCC: prognostic and diagnostic value

Given the severity and high recurrence rate of HCC, the prognosis remains poor. As a result, effective diagnostic and prognostic biomarkers are required. Several biomarkers have been suggested for HCC diagnosis and prognosis, including alpha-fetoprotein (AFP), Lens culinaris-agglutinin-reactive fraction of AFP (AFP-L3), protein induced by vitamin K absence orantagonist-II (PIVKA-II), vascular endothelial growth factor (VEGF), hypoxia-inducible factor (HIF), and others (53). On the other hand, circRNAs are increasingly linked to HCC progression and migration, despite their unknown function (54). Shang et al. discovered that hsa_circ_0005075 is overexpressed in HCC tissues and correlates with tumor size (55). Similarly, Qin et al. discovered that hsa_circ_0001649 was down expressed in HCC cells, indicating that it could be used as a prognostic and diagnostic biomarker (56). Recent studies have linked high ciRS-7 and AFP levels in HCC to hepatic microvascular invasion (57). CircRNAs could also communicate with miRNAs and act as miRNA sponges, influencing HCC progression by controlling downstream of a desired gene (58). Hsa_circ_0001649 and hsa_circ_0005075 contain potential miR-182 and miR-93 binding sites, respectively (59). MiR-182 and miR-93 have been identified as possible biomarkers for HCC prognosis and diagnosis. Furthermore, mounting evidence suggests that Wnt-related circRNAs have diagnostic biomarker potential and are linked to tumor development (60). In the following sections, we discuss about Wnt signaling pathways and their relationship with HCC as well as the role of the circRNA/Wnt axis in the HCC progression.

## 5 Interactions between circRNAs and Wnt signaling pathway in HCC

**Figure 2:**
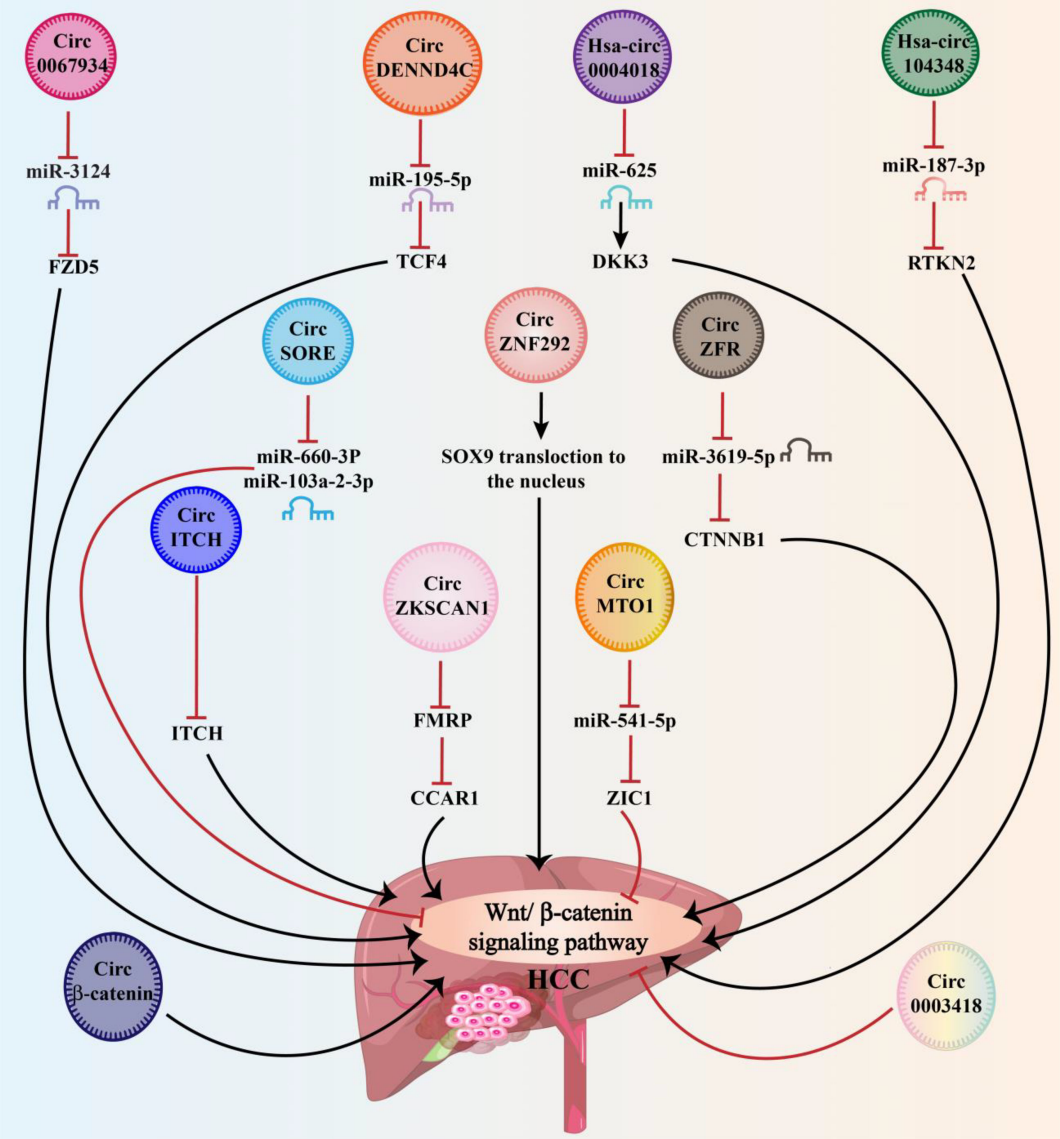
By sponging miRNAs, circRNA molecules regulate the Wnt signaling pathway for further modulation of HCC development in a positive and/or negative manner.

CircRNAs have been demonstrated to modulate cell proliferation, migration, and apoptosis by regulating gene transcription or by activating or inactivating signaling pathways associated with cancer (60). The most circRNAs function as miRNA sponges, activating or inhibiting the Wnt signaling pathway (61). As research advances, more circRNA-Wnt pathway interactions will be discovered. CircRNAs can influence cancer development and advancement through direct or indirect interactions with the Wnt pathway. Wnt-related circRNA abnormal activation has been linked to cancer cell proliferation, progression, invasion, and metastasis in tumors of the gastrointestinal and respiratory tracts, the central nervous system, the musculoskeletal system, and the endocrine system (60). The relevant studies suggest that this abnormal expression could be used as prognostic and diagnostic biomarkers, as well as treatment targets of cancer. These findings add to our knowledge of the fundamental processes that lead to cancer initiation and development. As a result, an overview of the most recent research on the circRNA-Wnt/β-catenin pathway in HCC and its regulatory framework for developing new treatments was conducted (**Figure 2** and **Table 1**). The next section will discuss the expression, characteristics, activities, and processes of circRNAs/Wnt axis in HCC.

**Table 1:** CircRNAs which are involved in the progression of the HCC through regulating Wnt signaling pathway.

CircRNA	Expression status in HCC	Targeted miRNA	Possible mechanism	Regulation of Wnt/β-catenin	Function	Ref
Circ-DENND4C	Increased	miRNA-195-5p	TCF4 overexpression	Activation	Facilitate the proliferation, invasion and stemness of HCC cell	(62)
Circ_0067934	Increased	miR-1324	FZD5 overexpression	Activation	Induce proliferation, migration, and invasion of HCC cells Inhibit the apoptosis	(63)
hsa_circ_0004018	Decreased	miR-625	Increased the transcription of DKK3 gene	Suppression	Inhibit the proliferation and migration of HCC cells	(64)
hsa_circRNA_104348	Increased	miR-187-3p	Increased the level of RTKN2	Activation	Induce the proliferation, invasion of HCC cells Suppress the apoptosis	(65)
CircRNA-SORE (circRNA_104797)	Increased	miR-660-3p and miR-103a-2-5p	No investigated	Activation	Induce the sorafenib resistance	(66)
CircZFR	Increased	miR-3619-5p	Increased CTNNB1 expression	Activation	Facilitate the proliferation and progression of HCC	(67)
CircRNA-ITCH	Decreased	miR-7 or miR-214	c-Myc and cyclin D1 oncogenes suppression	Suppression	Suppress the proliferation Increase the apoptosis	(68)
Circβ-catenin (circRNA-0004194)	Increased	No investigated	Encoded a novel β-catenin isoform	Activation	Facilitate proliferation, migration, invasion and metastasis of HCC cells	(69)
CircZKSCAN1 (hsa_circRNA_0001727)	Decreased	No investigated	Diminished the expression of CCAR1 by sponging FMRP	Suppression	Suppress cell stemness, proliferation, and metastasis in HCC	(70)
CircZNF292	Increased	No investigated	Increased β-catenin, p-STAT3, p-STAT5, Cyclin A, CDK2 Bind to SOX9 protein	Activation	Promote HCC cells proliferation and vascularization in the tumor environment Silencing CircZNF292 causes cell cycle arrest in G1 phase Silencing of this circRNA facilitated apoptosis	(71)
circMTO1 (hsa_circRNA_0007874)	Decreased	miR-541-5p	ZIC1 overexpression Regulating EMT pathway	Suppression	Inhibit the proliferation, migration, and invasion of HCC cells	(72)
		miR-9-5p	NOX4 overexpression	No investigated	Inhibited proliferation and migration of hepatoma cells Promoted apoptosis	(73)

MiR, microRNA; TCF4, Transcription factor 4; FZD5, Frizzled Class Receptor 5; DKK3, Dickkopf 3; RTKN2, Rhotekin 2; CCAR1, Cell Cycle and Apoptosis Regulator 1; FMRP, fragile X mental retardation protein; ZIC1, ZIC1 finger of cerebellum 1; NOX4, NADPH oxidase.

### 5.1 Circ_0067934

Circ_0067934 is a one type of circRNAs which has been indicated that participated in the HCC, esophageal squamous cell carcinoma, thyroid tumors, and lung cancer (74). Circ_0067934 overexpression is positively linked to the low overall survival of HCC. Zhu et al. discovered that silencing circ_0067934 reduced the cancer cells expansion, invasion, and metastasis, while enhancing the apoptosis both *in vitro* and *in vivo* (63). MiRNAs are generally known as regulators of various gene expression through targeting the mRNAs (75). It has been seen that silencing circ_0067934 considerably elevated miR-1324 levels in HCC cells. MiR-1324 overexpression suppresses the production of FZD5 protein which positively activates the Wnt/β-catenin signaling pathway. This pathway has a critical role in the HCC formation and progression (63, 76–78). In result, circ_0067934 could enhance FZD5 protein by inhibition of miR-1324 that leads to Wnt/β-catenin axis activation in HCC.

### 5.2 Circ_0003418

Circ_0003418 expression was discovered to be inversely related to tumor size, TNM stage, and HBsAg level in HCC cell lines. Circ_0003418 may reduce the ability of HCC cells to proliferate, migrate, and invade. Moreover, circ_0003418 reduced resistance to cisplatin in HCC cells by inhibiting the Wnt/β-catenin signaling pathway (79, 80)

### 5.3 CircRNA-ITCH

ITCH, an E3 ubiquitin protein ligase, is a protein which inhibits the Wnt/β-catenin signaling pathway. CircRNA-ITCH could increase the ITCH gene expression, indirectly inhibiting the Wnt/β-catenin signaling pathway (81, 82). CircRNA-ITCH played as an inhibitory factor for esophageal squamous cell carcinoma, lung cancer and triple-negative breast cancer by suppressing Wnt pathway (83–85). Furthermore, overexpression of circRNA-ITCH in HCC cell lines could substantially decrease HCC cell growth and enhance the cell death. CircRNA-ITCH could hinder the Wnt/β-catenin signaling pathway, resulting in a decrease in c-Myc and cyclin D1 oncogenes as Wnt/β-catenin pathway target genes (68, 86).

### 5.4 Circ−DENND4C

Circ-DENND4C (DENN domain containing 4C) has been shown to be overexpressed in breast cancer and glioma (87, 88). Liu et al., indicated that circ-DENND4C was significantly upregulated in HCC. They observed that silencing circ-DENND4C decreased the cell cycle-related proteins (Cyclin D1, CDK4) and Bcl-2, while enhancing Bax expression (62, 89). Moreover, they revealed that circ-DENND4C lowered the expression of miRNA-195-5p in HCC, which contributes to the overexpression of transcription factor 4 (TCF4). TCF4 overexpression resulted in the accumulation of β-catenin by activating the Wnt/β-catenin signaling pathway (62).

### 5.5 Has circ0004018

The expression of hsa_circ_0004018 was decreased in HCC compared with non-tumor tissue (90). According to reports, hsa_circ_0004018 serves as a sponge for miR-625. MiR-625 could target Dickkopf-3 (DKK3), a crucial gene in the hindering Wnt/β-catenin signaling pathway, and thus inhibit DKK3 gene expression. Hsa_circ_0004018 could suppress the HCC cell growth and invasion by diminishing miR-625 function, enhancing DKK3 gene expression and inactivating Wnt/β-catenin signaling pathway (64).

### 5.6 CircZNF292

Previous studies have indicated that circRNA zinc finger protein 292 (circZNF292) was upregulated in the hypoxic environment in the solid tumors, including HCC. CircZNF292 has proangiogenic function in the hypoxic condition of tumor environment *in vitro* (91–93). It has been demonstrated that circZNF292 knocking-down could decrease the downstream gene expression involved in Wnt/β-catenin signaling pathway including β-catenin, cyclin D1, and c-Myc. So, circZNF292 could promote the proliferation of HCC cells via Wnt/β-catenin signaling pathway activity (94). It has been shown that Wnt/β-catenin signaling pathway contributes to the vascularization in the tumor environment through upregulation of β-catenin which directly associated with the expression of the epithelial mesenchymal transition (EMT) factors, such as Twist1 and VE-cadherin (95, 96). CircZNF292 silencing significantly decreased β-catenin, Twist1 and VE-cadherin levels in the HCC cells through reducing Wnt/β-catenin signaling pathway activity. CircZNF292 could bind to SOX9 protein, a nuclear transcription factor which negatively regulates the activity of Wnt/β-catenin signaling pathway, in the cytoplasm and inhibited its translocation to the nucleus, resulting in the enhancement of Wnt/β-catenin signaling pathway activity and induction of vascularization (94). Thus, circZNF292 silencing could be investigated as a novel therapeutic strategy in HCC patients. In addition, knocking down circZNF292 caused cell cycle arrest in G1 phase, induced apoptosis and inactivated the Wnt/β-catenin signaling pathway of HCC cells, making circRNA ZNF292 as a target in HCC (71).

### 5.7 Hsa_circRNA_104348

It has been revealed that hsa_circRNA_104348 expression increased in the HCC and correlated with the poor prognosis. Overexpression of hsa_circRNA_104348 induced proliferation, whereas inhibiting apoptosis of the HCC cells (65). Rhotekin 2 (RTKN2) is a protein which is expressed in various tissues, but it has been reported that RTKN2 is upregulated in some cancers such as ovarian cancer, bladder cancer and HCC (97–100). The expression of RTKN2 can be regulated by miR-187-3p. Hsa_circRNA_104348 could act as a sponge for miR-187-3p and sequestered miR-187-3p, which contributed to RTKN2 overexpression in HCC. In addition, hsa_circRNA_104348 activated the Wnt/β-catenin signaling pathway and facilitated HCC cell proliferation, invasion, and metastasis (65).

### 5.8 CircRNA-SORE (circRNA_104797)

Sorafenib is a kinase inhibitor drug which is used in the advanced HCC (101). CircRNA-SORE has been shown to significantly increase the Sorafenib resistance by hindering the tumor cell apoptosis. circRNA-SORE play a role in sponging miR-660-3p and miR-103a-2-5p. MiR-660-3p and miR-103a-2-5p could significantly inhibit Wnt/β-catenin signaling pathway. Therefore, Sorafenib resistance is maintained by circRNA-SORE, which regulates the Wnt/β-catenin signaling pathway (66).

### 5.9 CircZFR

Circular RNA circZFR has been linked to the progression of HCC, breast cancer, papillary thyroid carcinoma, and bladder cancer (102–105). CircZFR functions as an oncogene in HCC, is significantly upregulated in HCC, and is associated with a poor prognosis in HCC patients. CircZFR has been shown to downregulate miR-3619-5p, while catenin Beta 1 (CTNNB1) expression is positively correlated with circZFR overexpression (106). CTNNB1 mutations were found in 18% to 40% of HCC patients and were associated with activation of the Wnt/β-catenin signaling pathway (62, 90). Thus, by regulating the miR-3619-5p/CTNNB1 axis, circZFR activated the Wnt/β-catenin signaling pathway, allowing circZFR silencing as a novel therapeutic target for HCC (106).

### 5.10 Circβ-catenin (circRNA-0004194)

CircRNA-0004194, also known as circβ-catenin, was overexpressed in the HCC. Liang et al., observed that circβ-catenin silencing *in vivo* and *in vitro* could repress the progression, migration, and invasion of HCC cells. Interestingly, circβ-catenin is a translatable protein that has resulted in an isoform known as β-catenin-370aa. This isoform could attenuate glycogen synthase kinase 3β (GSK3β) function through prevention of GSK3β binding to fill-length β-catenin. GSK3β inhibits β-catenin activity by β-catenin degradation. Thus, circβ-catenin could enhance β-catenin level in the tumor microenvironment by preventing β-catenin destruction, inducing Wnt/β-catenin signaling pathway (69, 107).

### 5.11 CircZKSCAN1 (hsa_circRNA_0001727)

The studies have shown that the expression of circZKSCAN1 was decreased in the HCC cell lines in comparison to the non-tumor tissue (70). Previous research reported that Fragile X mental retardation Protein (FMRP), a protein which mainly functions in the nervous system by targeting mRNAs, is overexpressed in HCC cells (108). CircZKSCAN1 and FMRP play opposing roles in HCC cells, with circZKSCAN1 suppressing stemness and FMRP increasing stemness through regulation of cell cycle and apoptosis regulator 1 (CCAR1) expression (70). CCAR1 is a β-catenin-binding protein that facilitates β-catenin in triggering Wnt target gene transcription (109–111). Moreover, CCAR1 overexpression in HCC is correlated to low survival rate (112). CircZKSCAN1 served as an RBP sponge in competition with target gene of FMRP, CCAR1, suppressing the Wnt/β-catenin signaling pathway, inhibiting cell stemness, expansion, and invasion in HCC and could be considered a therapeutic agent for HCC (70).

### 5.12 CircMTO1 (hsa_circRNA_0007874)

Previous studies were stated that circMTO1 play a role as a tumor suppressor (113) and inhibit the development of several cancers such as glioblastoma (114), gallbladder cancer (115), lung adenocarcinoma (116). The expression of circMTO1 was found to be downregulated in HCC. CircMTO1 could serve as a sponge for miR-541-5p and suppress its regulatory function. It has been demonstrated that ZIC1 is a direct target of miR-541-5p, as miR-541-5p expression correlated negatively with ZIC1 expression. Evidence has demonstrated that ZIC1 downregulation led to the significantly enhancement in β-catenin, cyclin D1, and c-Myc levels. In addition, an *in vivo* model, intratumoral injection of a miR-541-5p inhibitor reduced tumor size. As a result, the circMTO1/miR-541-5p/ZIC1 axis may have therapeutic potential for HCC by suppressing HCC cell growth, invasion, and metastasis through regulation of the Wnt/β-catenin signaling pathway (72). CircMTO1 is also a molecular sponge for miR-9-5p and it has been shown that overexpression of circMTO1 significantly reduced miR-9-5p expression. MiR-9-5p downregulation contributed to the upregulation of NOX4, a protein involved in the hepatocytes proliferation during physiological condition and tumorigenesis (73). Downregulation of NOX4 is associated with the tumorigenic potential and HCC formation (117, 118) and the activation of circMTO1/miR-9 5p/NOX4 axis promoted the apoptosis of HCC cells (73).

## 6 Wnt-related circRNA, a potential biomarker for cancer prognosis and treatment

Clinicians have believed that treatment choices require early prognostic data. A growing body of evidence suggests that Wnt-related circRNAs could be valuable for prognosis (60). In several malignancies, these circRNAs have a strong correlation with 5-year overall survival, disease-, recurrence-, and progression-free survival. According to Li et al., there was a negative correlation between the overexpression of circCCT3 and the colorectal cancer patient’s chance of disease-free survival (119). It has also been demonstrated that lower recurrence-free survival is associated with reduced circZKSCAN1 expression in HCC patients (70). Furthermore, increased level of circ_0109046 in endometrial cancer patients can predict a worse 5-year overall survival (120).Based on studies, it has been determined that circ_0067934 and circZFR are potential prognostic biomarkers in patients with HCC (63, 106). Such research has crucial consequences for determining prognosis and selecting treatments. Wnt-related circRNAs are also linked to other important prognostic variables. For instance, low levels of circMTO1 can predict late TNM stage and lymphatic metastases in colorectal cancer (CRC) (121). Thus, targeting Wnt-related circRNA expression could be a beneficial cancer therapeutic strategy. The most circRNAs function as miRNA sponges, activating or inactivating the Wnt pathway. It is also possible to control the target miRNAs of Wnt-related circRNAs. In CRC, for example, injection of MiR-582 significantly reversed the cellular mechanisms governed by circ_0009361 (122). CircRNA_NEK6 acts as a sponge for miR-370-3p, stimulating the FZD8/Wnt axis to promote the development of thyroid carcinoma (123). Circ_0121582 acts as a sponge of miR-224 in AML to overexpress GSK3β and trigger the Wnt/β-catenin pathway (124). Circ-SFMBT2 regulates the miR-1276/CTNNB1/Wnt/β-catenin axis, contributing to gastric cancer formation and progression. Knocking down circ_SMAD4 could inhibit the development of gastric cancer by decreasing cell proliferation (125). In addition, circ-ZNF124 silence has been shown to suppress NSCLC phenotypes (125). However, the current challenge is to identify targeted therapeutics capable of continually modulating circRNA expression and delivering its impact. This necessitates an in-depth knowledge of the biology and role of Wnt-related circRNAs.

## 7 Conclusions and future perspectives

NcRNAs are play a promising role in regulating different types of cancers includes HCC through the regulation of gene expression and modulation of biological pathways. In this context, targeting ncRNAs, include circRNAs, represents a novel method in cancer treatment and control drug resistance with advantages compared to conventional therapeutic strategies, such as specificity and potential reduction in toxicity (126, 127). For instance, Yi Li et al. proposed a potential approach to target circ_0000098 in HCC through a targeted drug delivery strategy using platelet-coated particles entrapped with doxorubicin and short hairpin RNA against circ_0000098. This was injected into mouse HCC models, and the results demonstrated remarkable sensitivity of HCC cells to doxorubicin and a reduction in drug resistance (127).

The Wnt signaling pathway is required for several physiological processes, including cell differentiation, motility, expansion, and muscular tissue formation. Both circRNA and the Wnt signaling pathway play important roles in cancer initiation and development. Developing evidence suggests that the circRNA/Wnt axis regulates tumor advancement, invasion, and metastasis by modulating the expression of cancer-associated genes. CircRNAs that are involved in the Wnt pathway typically perform their function by sponging miRNAs, which can be used as diagnostic markers. Wnt-related circRNAs may potentially be useful as biomarkers in the therapy of HCC. To manage tumor growth, researchers are attempting to either upregulate tumor-promoting circRNAs or downregulate tumor-suppressor circRNAs. Further research is needed to confirm the interactions and related mechanisms between suppressive miRNAs and corresponding circRNAs involved in the Wnt pathway for the diagnosis, prognosis, and treatment of HCC.

## Author contributions

The authors confirm contributions to this paper as follows,

AM, conceptualization, investigation, database searching, writing, and review manuscript. FZ, ST, HR, MM, YG, NH, SH, SG, RM, BG, and ZA, data collection, writing, draft preparation, and figure design. MA and HM contributed in the project administration, editing, validation, coordinated the study, and accurately evaluated the manuscript. All authors contributed to the article and approved the submitted version.
